# LncRNA *NEAT1* Knockdown Inhibits Retinoblastoma Progression by miR-3619-5p/LASP1 Axis

**DOI:** 10.3389/fgene.2020.574145

**Published:** 2020-11-17

**Authors:** Xuedong Chen, Shiyong Zhao, Qingjun Li, Caicai Xu, Yongbin Yu, Hongyan Ge

**Affiliations:** ^1^Department of Ophthalmology, The First Affiliated Hospital of Harbin Medical University, Harbin, China; ^2^Department of Hepatopancreatobiliary Surgery, The Second Affiliated Hospital of Harbin Medical University, Harbin, China

**Keywords:** retinoblastoma, lncRNAs, *NEAT1*, miR-3619-5p, LASP1

## Abstract

Retinoblastoma (RB) is the most common intraocular tumor in childhood. Long non-coding RNA (lncRNA) nuclear paraspeckle assembly transcript 1 *(NTAT1)* has been reported to be related to RB progression. This study aims to study the molecular mechanism of *NEAT1* in regulating cell cycle, proliferation, apoptosis, migration, and invasion in RB. The expression levels of *NEAT1* and miR-3619-5p were detected by quantitative real-time polymerase chain reaction (qRT-PCR). The protein expression of LIM and SH3 domain protein 1 (LASP1) was measured by western blot. The proliferation of RB cells was analyzed by cell counting kit-8 (CCK-8) and cell colony formation assays. Cell migration and invasion were evaluated by transwell assay. Cell cycle and apoptosis were assessed by flow cytometry analysis. The association between miR-3619-5p and *NEAT1* or LASP1 was predicted by starBase 3.0 database and identified by dual-luciferase reporter assay. The effects of *NEAT1* knockdown on the tumor growth *in vivo* were detected by *in vivo* tumor formation assay. *NEAT1* expression was dramatically up-regulated, and miR-3619-5p expression was obviously downregulated in RB tissues and cells compared with control groups. The protein level of LASP1 was obviously increased in RB tissues or cells relative to paracancerous normal tissues or cells, respectively. Functionally, *NEAT1* silencing inhibited RB cell migration, invasion, and proliferation, whereas induced cell apoptosis and cell cycle arrest in RB; this phenomenon was partially abolished by miR-3619-5p inhibitor. Mechanistically, *NEAT1* acted as a sponge of miR-3619-5p, and miR-3619-5p was associated with LASP1. In addition, *NEAT1* knockdown decreased the volume and weight of RB tumor *in vivo*. Together, *NEAT1* silencing repressed cell migration, invasion, and proliferation, whereas induced cell apoptosis and cycle arrest by sponging miR-3619-5p to inhibit LASP1 expression in RB cells. This study may provide a theoretical basis for RB therapy.

## Introduction

Retinoblastoma (RB) is the most common intraocular tumor in childhood. It has been reported that 90% RB patients are under 5 years old, and 20–30% cases are neonates (Mehyar et al., 2020). The survival rate of RB at the early stage is about 100%. However, advanced RB can lead to death owing to its metastases from brain to tissues (Villegas et al., 2013). Although much effort and progress have been done, the pathogenesis of RB progression is still fuzzy.

Long non-coding RNAs (LncRNAs) are a class of transcripts > 200 nucleotides in length (Li et al., 2015). LncRNAs regulate gene expression at transcriptional and post-translational levels. LncRNA dysregulation is related to cancer progression, including RB (Li J. et al., 2016; Sheng et al., 2018) Nuclear paraspeckle assembly transcript 1 (*NEAT1*) also participates in RB progression. Wang et al. indicated that *NEAT1* silencing repressed cell proliferation and promoted apoptosis by inhibiting miR-124 in RB (Wang L. et al., 2019). Zhong et al. (2019) investigated that *NEAT1* knockdown suppressed cell metastasis, whereas promoted cell apoptosis by associating with miR-204 to inhibit CXC chemokine receptor 4 (CXCR4) expression in RB. In this study, the regulatory mechanism of RB progression by *NEAT1* was further explored.

MicroRNAs (miRNAs) are 18–22 nucleotides in length. And miRNAs work via regulating the 3′ untranslated region (3′UTR) of target genes, which results in their mRNA degradation and the repression of protein translation (Hua et al., 2011). MiR-3619-5p is also associated with cancer development. Niu et al. (2015) suggested that miR-3619-5p inhibited lung cancer progression by modulating β-catenin 3′UTR. Zhang et al. (2018) indicated that miR-3619-5p repressed cell metastasis by inhibiting beta-catenin, cyclin-dependent kinase 2 (CDK2) and activating p21 in bladder carcinoma. In addition, CDKN1A-mediated miR-3619-5p was also reported to repress cell proliferation and promote cell apoptosis in prostate cancer (Li et al., 2017). However, the mechanism by which miR-3619-5p affects RB growth is still poorly understood.

LIM and SH3 domain protein 1 (LASP1) belongs to LIM family and plays a vital part in cell structure with acting as transmitting messages from cytoplasm to nucleus (Orth et al., 2015). LASP1 is indicated to be overexpressed in various cancers (Liu H. et al., 2019). Zheng et al. (2016) suggested that LASP1 silencing hindered cell proliferation and metastasis in esophageal cancer (Liu H. et al., 2019). Shimizu et al. (2013) investigated that LASP1 silencing induced cell cycle accumulation in G2 phase in oral cancer. In addition, LASP1 silencing inhibited cell migration in clear cell renal cell cancer (Yang et al., 2014). But there are few studies on the regulation of RB progression by LASP1.

In this study, *NEAT1* expression was determined by quantitative real time polymerase chain reaction (qRT-PCR). Mechanistically, loss-of-function experiments were carried out to determine the impacts of *NEAT1* on RB progression. Functionally, the association among *NEAT1*, miR-3619-5p and LASP1 was predicted by starBase 3.0 database and identified by dual-luciferase reporter assay. In addition, *in vivo* tumor formation assay was used to reveal the impacts of *NEAT1* knockdown on RB progression *in vivo*.

## Materials and Methods

### Clinical Tissue Sample and Cell Culture

Forty pairs of RB tissues and paracancerous normal tissues (2 cm far from tumors) were collected during biopsy at The First Affiliated Hospital of Harbin Medical University and instantly placed in liquid nitrogen. The Ethics Committee of The First Affiliated Hospital of Harbin Medical University allowed this study. RB patients signed the written informed consents.

BeNa Culture Collection (Beijing, China) supplied the human RB cell lines (Y-79 and SO-RB50) and human retinal epithelial cell ARPE-19. All cells were stored in liquid nitrogen and cultivated in RAPI-1640 medium (HyClone, Logan, UT, United States) containing 10% fetal bovine serum and 1% penicillin-streptomycin (HyClone) at 37°C in a humid condition with 5% CO_2_.

### Cell Transfection

Small interfering RNA targeting *NEAT1* (si-NEAT1), the overexpression plasmid of NEAT1 (*NEAT1*), miR-3619-5p mimic, miR-3619-5p inhibitor, the overexpression vector of LASP1 (pc-LASP1), small hairpin RNA against *NEAT1* (sh-*NEAT1*) and their control groups, siRNA negative control (si-NC), vector, miRNA NC, inhibitor NC, pc-NC and sh-NC, were purchased from GenePharma (Shanghai, China). si-*NEAT1* and si-NC were used to determine the interfering efficiency of si-*NEAT1*. In order to verify the relationship among *NEAT1*, miR-3619-5p and LASP1, the si-*NEAT1*, miR-3619-5p mimic/inhibitor or pc-LASP1 was transfected into cells with control groups. Cell transfection was carried out using Lipofectamine 2000 reagent (Thermo Fisher, Waltham, MA, USA) according to the manufacturer’s instructions. The primer sequences were si-*NEAT1* 5′-GGAGGAGTCAGGAGGAATA-3′; si-NC 5′-GGATGAGACGAGAGGGATA-3′; miR-3619-5p mimic 5′-UCAGCAGGCAGGCUGGUGCAGC-3′; miRNA NC: 5′-UUUGUACUACACAAAAGUACUG-3′; miR-3619-5p inhibitor 5′-GCUGCACCAGCCUGCCUGCUGA-3′ and inhibitor NC 5′-CAGUACUUUUGUGUAGUACAAA-3′.

### RNA Extraction and qRT-PCR

RB tissues and cells were lysed using RNAiso Plus (TaKaRa, Dalian, China). Then, RNA was extracted with RNAprep Pure Tissue kit (Tiangen, Beijing, China). The cDNA was amplified by a reagent kit (TaKaRa). A SYBR^®^ Premix DimerEraser Kit (TaKaRa) was utilized to detect the expression levels of *NEAT1*, miR-3619-5p and LASP1. U6 snRNA and GAPDH were utilized to normalize the miRNA and lncRNA/mRNA. The sense and anti-sense primers were: *NEAT1* 5′-CTTCCTCCCTTTAACTTATCCATTCAC-3′ and 5′-CTCTTCCTCCACCATTACCAACAATAC-3′; miR-3619-5p 5′-TCATCAGCAGGCAGGCTGGTGC-3′ and 5′-GTGCAGGG TCCGAGGT-3′; LASP1 5′-CTGTCTCTGCCTTATAGCAACAC-3′ and 5′-CATCTCGAA CCTGGCTGTTTG-3′; U6 5′-TGCG GGTGCTCGCTTCGGCAGC-3′ and 5′-GTGCAGGGTCCGAG GT-3′; GAPDH 5′-TATGATGATATCAAGAGGGTAGT-3′ and 5′-TGTATCCAAACTCATTGTCATAC-3′.

### CCK-8 Assay

Y-79 and SO-RB50 cells were cultivated in 96-well plate for 24 h. Then, 10 μL CCK-8 was added into plate after cell transfection. Cells were continued to culture for 4 h and results were analyzed by measuring the absorbance at 450 nm with microscope reader.

### Transwell Assay

The invasion and migration abilities of Y-79 and SO-RB50 cells were determined by transwell chamber with or without Matrigel (Corning, Shanghai, China), respectively. Briefly, cells were suspended in medium without serum. Following cells were added in the upper chamber. Medium with 10% FBS was added into down chamber. Then cells were cultivated for 24 h, following supernatant was discarded. Cells were incubated with methanol for 30 min and crystal violet for 20 min. Results were visualized by microscope at a 100× magnification.

### Cell Colony Formation Assay

Cells were cultivated in 6-well plate (500 cells per well) for 2 weeks. The proliferating colonies were stained with 1% crystal violet. Then colonies numbers were calculated and results were analyzed. A colony was defined when its cell number more than 50.

### Flow Cytometry Assay

Cell cycle and apoptosis were analyzed by cell cycle and apoptosis detection kit (Beyotime, Shanghai, China). In short, Y-79 and SO-RB50 cells were harvested after digested with trypsin (Thermo Fisher Scientific), and cells were washed with cold phosphate buffered solution (PBS). Then, cells were suspended in PBS, following were centrifuged. Cells were subsequently fixed with 70% anhydrous ethanol for 30 min. Cells were centrifuged and supernatant was removed. Dye buffer, propidium iodide (PI) and RNase A were added and were incubated with cells at 37°C for 30 min. Samples were assessed by flow cytometry (BD Biosciences, San Diego, CA, United States).

### Dual-Luciferase Reporter Assay

StarBase 3.0 online database was employed to investigate the association between miR-3619-5p and *NEAT1* or LASP1. The wide-type (WT) *NEAT1* containing the binding sites of miR-3619-5p and mutant (MUT) *NEAT1* were amplified and inserted into the pGL3 vector (Promega Corporation, Fitchburg, WI, United States), named as WT-*NEAT1* and MUT-*NEAT1*. The WT-LASP1-3′UTR and MUT-LASP1-3′UTR were synthesized and inserted into the pGL3 vector, named as WT-LASP1-3′UTR and MUT-LASP1-3′UTR. Then, the plasmids were transfected into cells with miR-3619-5p mimic or miRNA NC using Lipofectamine 2000 reagent according to the manufacturer’s instructions. The results were detected by dual-luciferase reporter system. *Renilla* luciferase activity was chosen as a reference.

### Western Blot

Samples were treated with RIPA buffer (Thermo Fisher Scientific). Then the lysate was loaded on 10% SDS-PAGE. Then bands were transferred onto polyvinylidene fluoride membrane. Bands were blocked in the 5% not-fat milk. The membranes were incubated with primary antibodies. Subsequently, the membranes were washed and incubated with secondary antibody labeled with horseradish peroxidase. Protein bands were visualized by ECL system (Pierce, Rockford, IL, United States). GAPDH was used as a reference. The primary antibodies were anti-LASP1 (1:3000; Proteintech, Rosemount, MN, United States) and anti-GAPDH (1:4000; Proteintech).

### RNA Pull-Down Assay

Y-79 and SO-RB50 cells were grown for 16 h, si-NEAT1 and si-NC were severally transfected into cells according to the manufacturer’s instructions. 36 h later, biotinylated mi-3619-5p was transfected into Y-79 and SO-RB50 cells and cells were continued to culture 48 h. Following cells were collected and lysed using lysis buffer. Then lysates were incubated with Streptavidin-coupled Dynabeads (Invitrogen, Carlsbad, CA, United States) and proteinase K (Millipore, Billerica, MA, United States), respectively. The amount of ASAP1 was detected by qRT-PCR.

### *In vivo* Tumor Formation Assay

RB Cells (5 × 10^6^) transfected with sh-*NEAT1* or sh-NC were injected into six-week old nude mice. Then, mice were continued to feed for 7 days. The tumor volume was recorded every 7 days. All nude mice were sacrificed at 28th day after injection. The tumors were excised and weight was analyzed. This study was approved by Animal Care and Use Committee of The First Affiliated Hospital of Harbin Medical University.

### Statistical Analysis

Data analysis was carried out based on ≥ 3 independent experiments. All data were assessed by GraphPad Prism 5.0 (GraphPad Software, La Jolla, CA, United States) or mage J software (NIH, Bethesda, MD, United States). Data was represented as mean ± SD. The comparison in study was evaluated by Student’s *t*-test, one-way analysis of variance or chi-square test. *P* < 0.05 was considered statistically significant.

## Results

### *NEAT1* Expression Level Was Dramatically Upregulated in Retinoblastoma Tissues and Cells

**FIGURE 1:**
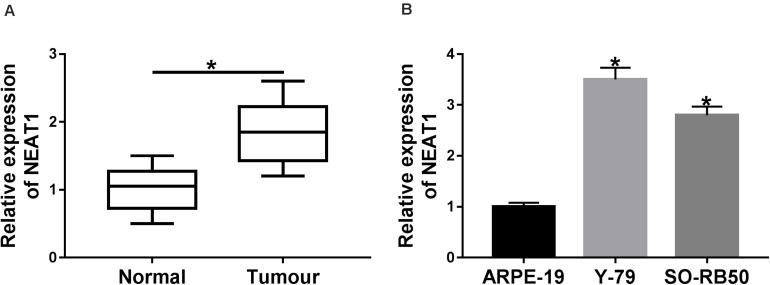
*NEAT1* expression level was dramatically up-regulated in RB tissues and cells. **(A)** The expression of *NEAT1* in RB tissues and adjacent normal tissues was detected by qRT-PCR. **(B)** *NEAT1* expression level in RB cells and ARPE-19 cells was assessed by qRT-PCR. *P < 0.05.

In order to determine the expression characteristics of *NEAT1* in RB tissues and cells, its expression was detected by qRT-PCR. Results showed that expression level of *NEAT1* was obviously upregulated in RB tissues compared with paracancerous normal tissues (Figure 1A). Additionally, qRT-PCR analysis revealed that the expression level of *NEAT1* was higher in both Y-79 and SO-RB50 cells than in ARPE-19 (Figure 1B). These data investigated that *NEAT1* was overexpressed in RB tissues and cells.

### *NEAT1* Regulated Cell Migration, Invasion, Proliferation, Cycle Arrest, and Apoptosis in RB

**FIGURE 2:**
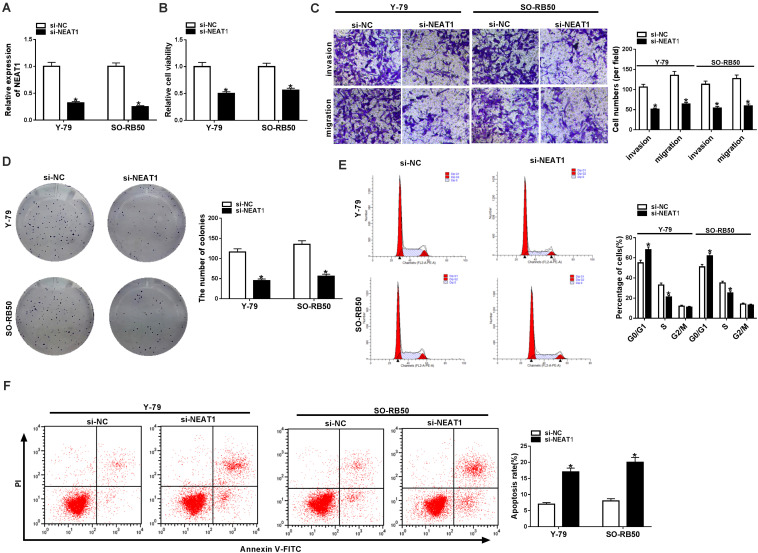
*NEAT1* silencing repressed the progression of Y-79 and SO-RB50 cells. **(A)** The knockdown efficiency of si-*NEAT1* in Y-79 and SO-RB50 cells was determined by qRT-PCR. **(B)** The effect of *NEAT1* knockdown on Y-79 and SO-RB50 cell viability was detected by CCK-8 assay. **(C)** The effects of *NEAT1* silencing on the invasive and migratory abilities of Y-79 and SO-RB50 cells were detected by transwell assay. **(D)** Cell colony formation assay showed that the colony-forming ability of Y-79 and SO-RB50 cells was repressed by *NEAT1* knockdown. **(E)** Flow cytometry analysis showed that cell cycle was arrested in G0/G1 phase by *NEAT1* silencing in Y-79 and SO-RB50 cells. **(F)** Flow cytometry assay showed that *NEAT1* knockdown induced RB cell apoptosis in Y-79 and SO-RB50 cells. *P < 0.05.

To explore the functional effects of *NEAT1* on RB cell progression, the silencing efficiency of si-*NEAT1* was firstly evaluated by qRT-PCR. Results showed that *NEAT1* was dramatically repressed after si-*NEAT1* transfection in Y-79 and SO-RB50 cells (Figure 2A). Then, the impacts of *NEAT1* knockdown on RB progression were determined. CCK-8 assay explained that the viability of Y-79 and SO-RB50 cells was inhibited by *NEAT1* knockdown (Figure 2B). Transwell assay demonstrated that *NEAT1* silencing inhibited the invasive and migratory abilities of Y-79 and SO-RB50 cells (Figure 2C). Cell colony formation assay showed that cell colony-forming ability was repressed by *NEAT1* downregulation in Y-79 and SO-RB50 cells (Figure 2D). In addition, flow cytometry analysis revealed that *NEAT1* knockdown induced cell cycle arrest in G0/G1 phase and apoptosis in Y-79 and SO-RB50 cells (Figures 2E,F). In order to further demonstrate that *NEAT1* acted as an oncogene in RB progression, the effects of *NEAT1* overexpression on the viability and apoptosis of normal retina cells (ARPE-19) were revealed. Results showed that ectopic *NEAT1* expression had no impact on cell viability and apoptosis (Supplementary Figures 1A,B). All data suggested that *NEAT1* contributed to RB development.

### *NEAT1* Knockdown Repressed RB Cell Progression by Sponging miR-3619-5p

**FIGURE 3:**
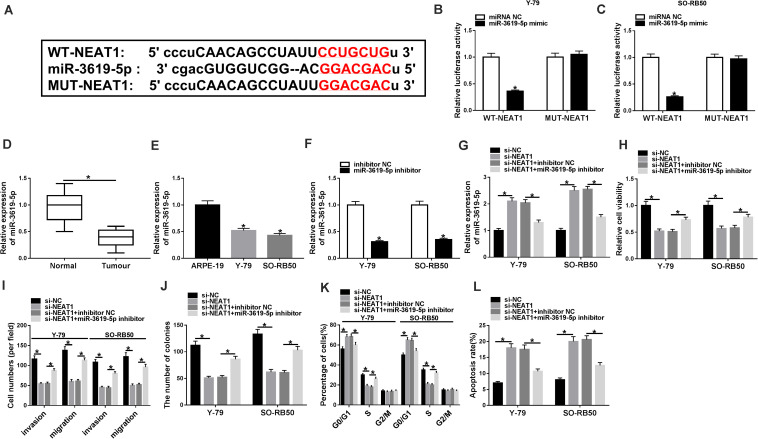
MiR-3619-5p inhibitor partially reversed the effects of *NEAT1* knockdown on RB progression. **(A)** The binding sequence of miR-3619-5p in *NEAT1* was predicted by starBase 3.0 database. **(B,C)** Dual-luciferase reporter assay detected luciferase activities in Y-79 and SO-RB50 cells. **(D,E)** MiR-3619-5p expression was determined by qRT-PCR in RB tissues and cells. **(F)** The silencing efficiency of miR-3619-5p inhibitor was determined by qRT-PCR. **(G)** The effects between *NEAT1* knockdown and miR-3619-5p inhibitor on miR-3619-5p expression in Y-79 and SO-RB50 cells were detected by qRT-PCR. **(H,J)** The cell proliferation was detected by CCK-8 and cell formation colony assay in Y-79 and SO-RB50 cells. **(I)** Transwell assay detected the migration and invasion of Y-79 and SO-RB50 cells. **(K,L)** Flow cytometry assay accessed the effects between *NEAT1* knockdown and miR-3619-5p inhibitor on cell cycle and apoptosis. *P < 0.05.

To understand the molecular mechanism of *NEAT1* knockdown in regulating RB cell development, the associated gene with *NEAT1* was predicted by starBase 3.0 database. Figure 3A showed that *NEAT1* contained the binding sites of miR-3619-5p. Further, dual-luciferase reporter assay showed that the luciferase activity of WT-*NEAT1* + miR-3619-5p group was greatly inhibited in Y-79 and SO-RB50 cells, whereas the luciferase activity of MUT-*NEAT1* + miR-3619-5p group had no significant change (Figures 3B,C). Subsequently, miR-3619-5p expression was detected by qRT-PCR in RB tissues and cells. Results showed that miR-3619-5p was downregulated in RB tissues and cells relative to control groups (Figures 3D,E). These outcomes elucidated that *NEAT1* was a sponge of miR-3619-5p.

In order to detect the effects between *NEAT1* knockdown and miR-3619-5p inhibitor on RB progression, the silencing efficiency of miR-3619-5p inhibitor was firstly determined by qRT-PCR. Results revealed that miR-3619-5p inhibitor obviously repressed the expression of miR-3619-5p (Figure 3F). Subsequently, qRT-PCR analysis suggested that *NEAT1* silencing up-regulated miR-3619-5p expression, whereas miR-3619-5p inhibitor partially reversed this effect (Figure 3G). CCK-8 and cell colony formation assays showed that *NEAT1* repression inhibited RB cell proliferation, whereas this phenomenon was partially abolished by miR-3619-5p inhibitor (Figures 3H,J). Transwell assay showed that cell invasive and migratory abilities were obviously repressed by *NEAT1* knockdown in Y-79 and SO-RB50 cells; however, the inhibition effect was decreased by miR-3619-5p inhibitor (Figure 3I). Further, flow cytometry analysis showed that *NEAT1* knockdown induced the cycle arrest and apoptosis of Y-79 and SO-RB50 cells, and miR-3619-5p inhibitor partly restored these effects (Figures 3K,L). These data indicated that *NEAT1* silencing regulated RB progression by binding to miR-3619-5p.

### LASP1 Overexpression Partially Attenuated the Effects of miR-3619-5p Mimic on the Cell Proliferation, Migration, Invasion, Cell Cycle and Apoptosis in RB

**FIGURE 4:**
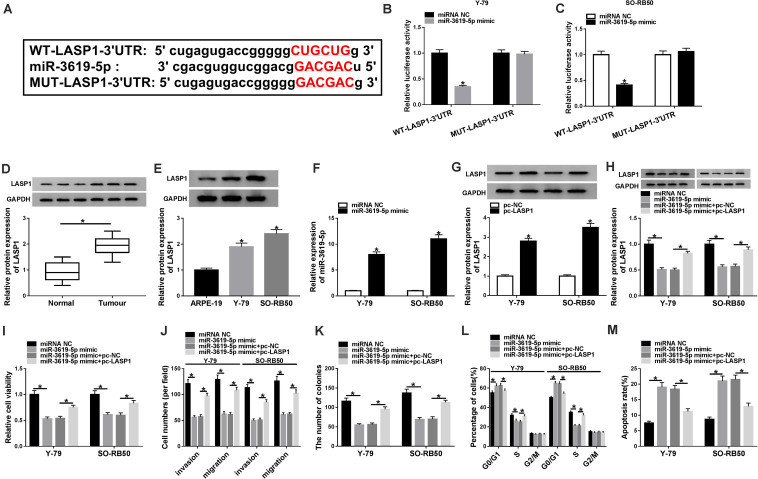
MiR-3619-5p repressed RB cell progression by sponging LASP1. **(A)** The starBase 3.0 database predicted the binding sites between miR-3619-5p and LASP1. **(B,C)** The luciferase activities were detected by dual-luciferase reporter assay. **(D,E)** The protein level of LASP1 was determined by western blot in RB tissue and cells. **(F)** The transfection efficiency of miR-3619-5p mimic was detected by qRT-PCR. **(G)** Western blot detected the transfection efficiency of pc-LASP1 in Y-79 and SO-RB50 cells. **(H)** The impacts between miR-3619-5p mimic and LASP1 overexpression on LASP1 expression were determined by western blot in Y-79 and SO-RB50 cells. **(I,K)** The cell proliferation was detected by CCK-8 and cell colony formation assays in Y-79 and SO-RB50 cells. **(J)** The invasive and migratory abilities of RB cells were determined by transwell assay in Y-79 and SO-RB50 cells. **(L,M)** Flow cytometry analysis revealed that the effects between miR-3619-5p mimic and LASP1 overexpression on the cell cycle and apoptosis of Y-79 and SO-RB50 cells. *P < 0.05.

In order to further determine that how miR-3619-5p regulated RB development, the targeting gene of miR-3619-5p was predicted. Results revealed that LASP1-3′UTR contained the binding sites of miR-3619-5p (Figure 4A). Subsequently, dual-luciferase reporter assay investigated that the luciferase activity of WT-LASP1-3′UTR + miR-3619-5p group was significantly decreased in both Y-79 and SO-RB50 cells; however, the luciferase activity of MUT-LASP1-3′UTR + miR-3619-5p group was not changed (Figures 4B,C). In addition, LASP1 protein expression was detected in RB tissues and cells, and western blot analysis showed that the protein level of LASP1 was significantly up-regulated in RB tissues or cells compared with normal tissues or cells, respectively (Figures 4D,E). These results suggested that miR-3619-5p targeted LASP1.

To investigate the effects between miR-3619-5p mimics and LASP1 overexpression on RB progression, the transfection efficiency of miR-3619-5p mimic and pc-LASP1 was firstly detected. The qRT-PCR analysis showed that miR-3619-5p mimic strongly increased the expression of miR-3619-5p (Figure 4F), and western blot assay explained that LASP1 protein level was apparently up-regulated after pc-LASP1 transfection (Figure 4G). Then western blot analysis explained that miR-3619-5p mimic inhibited LASP1 expression and this effect was decreased by LASP1 overexpression (Figure 4H). Based on the above data, CCK-8 and cell colony formation assay showed that miR-3619-5p mimic inhibited the viability and colony-forming ability of Y-79 and SO-RB50 cells, whereas LASP1 overexpression partly attenuated this inhibition effect (Figures 4I,K). Similarly, the cell invasive and migratory abilities of RB were repressed by miR-3619-5p mimic; however, this phenomenon was partially abolished by LASP1 overexpression (Figure 4J). In addition, miR-3619-5p mimic induced cell cycle arrest in G0/G1 phase and apoptosis in Y-79 and SO-RB50 cells, but these impacts were partly restored by pc-LASP1 (Figures 4L,M). Collectively, these results demonstrated that miR-3619-5p inhibited RB cell progression by targeting LASP1-3′UTR.

### *NEAT1* Knockdown Downregulated the Protein Expression of LASP1 by Sponging miR-3619-5p

**FIGURE 5:**
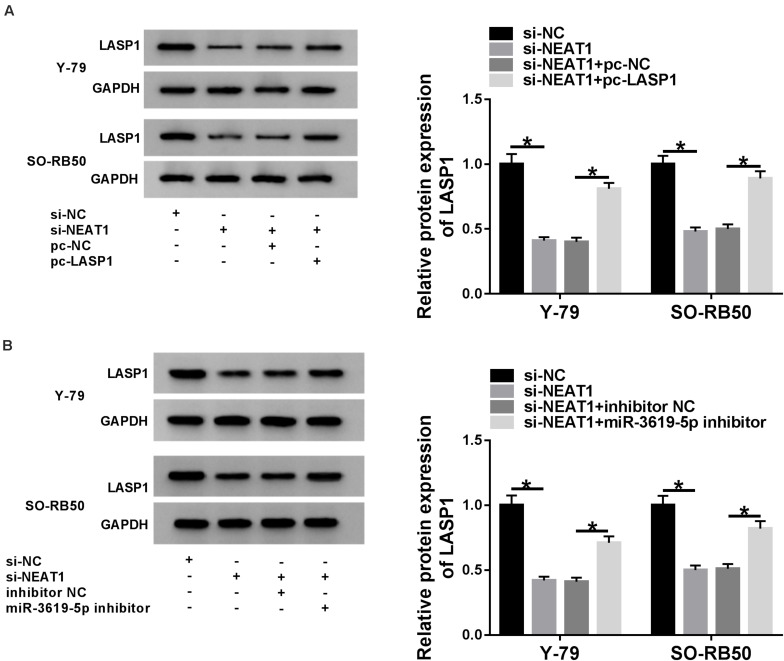
*NEAT1* silencing repressed the protein level of LASP1 by regulating miR-3619-5p. **(A)** The effects between *NEAT1* knockdown and LASP1 overexpression on LASP1 protein expression were revealed by western blot. **(B)** Western blot demonstrated that *NEAT1* knockdown repressed the protein expression of LASP1, which was decreased by miR-3619-5p inhibitor. *P < 0.05.

To further determine the relationship among *NEAT1*, miR-3619-5p and LASP1, the effects between *NEAT1* knockdown and LASP1 overexpression or miR-3619-5p inhibitor on LASP1 protein expression were detected in Y-79 and SO-RB50 cells. Western blot revealed that *NEAT1* silencing repressed LASP1 protein expression, whereas this effect was partially abolished by LASP1 overexpression or miR-3619-5p inhibitor (Figures 5A,B). In order to further illustrate whether NEAT1 affected LASP1 expression by sponging miR-3619-5p, RNA pull-down assay was employed. Results showed that the enrichment of LASP1 by miR-3619-5p was dramatically increased after NEAT1 silencing (Supplementary Figure 2). Thus, all data suggested that *NEAT1* up-regulated LASP1 expression by sponging miR-3619-5p.

### *NEAT1* Knockdown Repressed Rb Growth *in vivo*

**FIGURE 6:**
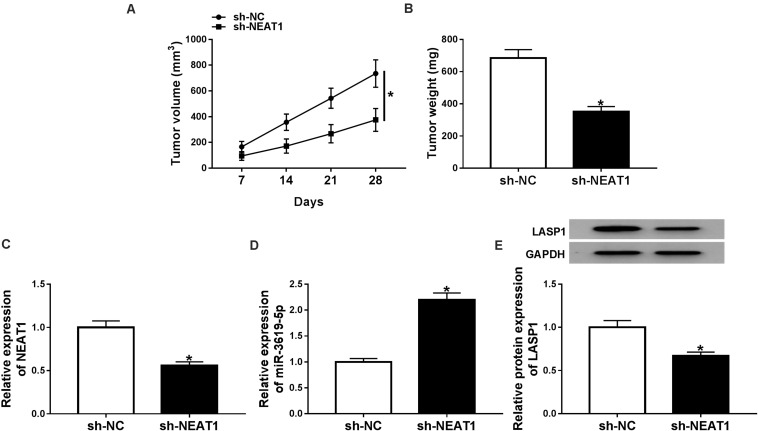
*NEAT1* knockdown inhibited RB growth *in vivo*. **(A)** Tumor volume was inhibited by *NEAT1* knockdown. **(B)** The weight of tumors was reduced after NEAT1 silencing *in vivo*. **(C)** The interfering efficiency of sh-*NEAT1* was determined by qRT-PCR. **(D)** QRT-PCR showed that *NEAT1* knockdown up-regulated miR-3619-5p expression *in vivo*. **(E)** Western blot analysis showed that *NEAT1* knockdown repressed the protein level of LASP1 *in vivo*. *P < 0.05.

The effects of *NEAT1* silencing on RB growth *in vivo* were explored. Results showed that tumor volume and weight were dramatically decreased by *NEAT1* silencing (Figures 6A,B). Further, the effects of *NEAT1* knockdown on miR-3619-5p expression and LASP1 protein level were analyzed *in vivo*. QRT-PCR revealed that the interfering vector of *NEAT1* was successfully built (Figure 6C) and *NEAT1* knockdown up-regulated miR-3619-5p expression (Figure 6D). Western blot analysis showed *NEAT1* silencing inhibited LASP1 protein level (Figure 6E). From these data, it was concluded that *NEAT1* knockdown inhibited RB growth by regulating miR-3619-5p and LASP1 *in vivo*.

## Discussion

LncRNAs were revealed to work by sponging miRNAs to repress mRNA expression (Dong et al., 2018). LncRNAs can be employed to as biomarkers for cancer treatment based on LncRNAs regulating cell development. LncRNA *NEAT1*, a scaffold factor, has been investigated to take part in diverse cancer progression.

Zhuang et al. (2020) indicated *NEAT1* was up-regulated in colon cancer and its overexpression promoted the metastasis of colon cancer cells. *NEAT1* was indicated to be overexpressed in pancreatic cancer and increased cell proliferation and metastases in pancreatic cancer cells (Feng et al., 2020). Pang et al. (2019) investigated that *NEAT1* expression was increased and *NEAT1* elevated cell cycle progression in breast cancer. In addition, Yu et al. (2019) suggested that *NEAT1* knockdown promoted cell apoptosis in lung cancer. Similarly, we found *NEAT1* expression was up-regulated and *NEAT1* knockdown inhibited cell proliferation, migration and invasion, and promoted cell cycle arrest and apoptosis in RB. Meanwhile, our data showed that *NEAT1* overexpression enhanced cell viability, whereas had no effect on cell apoptosis in normal retina cells (ARPE-19). These results indicated that *NEAT1* functioned as an oncogene in RB progression.

MiR-3619-5p was indicated to repress the proliferation and metastasis of RB cells (Yan et al., 2019). In addition, miR-3619-5p promoted cell apoptosis in papillary thyroid carcinoma (Wang G. et al., 2019). Li et al. (2017) explained that miR-3619-5p promoted cell cycle arrest in prostate cancer cells. Coincidentally, our study showed that *NEAT1* was a sponge of miR-3619-5p. MiR-3619-5p was revealed that its overexpression repressed cell proliferation, migration and invasion, and promoted cell cycle arrest and apoptosis in RB. These data were consistent with previous results. Besides, miR-3619-5p expression was downregulated in RB tissues and cells. Collectively, all data suggested that *NEAT1* promoted RB development by sponging miR-3619-5p.

Further, we found miR-3619-5p targeted LASP1 and inhibited LASP1 expression, and LASP1 could decrease the inhibition effects of miR-3619-5p on RB progression. All the above results suggested that LASP1 had promotion effects on RB progression. Liu H. et al. (2019) indicated that the inhibition effect of miR-143 on cell metastasis was impaired by LASP1 in human esophageal cancer. In esophageal cancer, circ-0004370 silencing suppressed cell proliferation and induced cell apoptosis, whereas these effects were decreased by LASP1 (Zhang et al., 2019). Our results were similar to these findings. In addition, our studies suggested that LASP1 inhibited cell cycle accumulation in G0/G1 phase. Nevertheless, LASP1 was found to induce cell cycle arrest in G2/M phase in thyroid cancer and gallbladder cancer (Li Z. et al., 2016; Liu W. et al., 2019). The reason for this phenomenon maybe was that the regulatory mechanism of LASP1 in cell cycle was discrepant in various cancers. The finding in this part investigated that miR-3619-5p inhibited RB progression by targeting LASP1.

Taken together, *NEAT1* and LASP1 expression were dramatically up-regulated, and miR-3619-5p expression was obviously downregulated in RB tissues and cells. *NEAT1* knockdown inhibited cell proliferation and metastasis, whereas promoted cell apoptosis and cell cycle arrest in RB. Enforced *NEAT1* expression fas had no effect on cell viability and apoptosis in normal retina cells. MiR-3619-5p inhibitor decreased the inhibition effects of *NEAT1* knockdown on RB progression. *NEAT1* was a sponge of miR-3619-5p and miR-3619-5p was associated with LASP1. Besides, *NEAT1* knockdown repressed RB growth *in vivo*. Therefore, we came into a conclusion that *NEAT1* silencing inhibited RB cell progression by downregulating LASP1 expression through sponging miR-3619-5p as showed in Supplementary Figure 3, which established a foundation for further study of RB progression and provided a theory evidence in studying RB therapy.

## Data Availability Statement

The original contributions presented in the study are included in the article/Supplementary Material, further inquiries can be directed to the corresponding author/s.

## Ethics Statement

The studies involving human participants were reviewed and approved by The First Affiliated Hospital of Harbin Medical University. The patients/participants provided their written informed consent to participate in this study. The animal study was reviewed and approved by The First Affiliated Hospital of Harbin Medical University.

## Author Contributions

HG designed and supervised the study. XC conducted the experiments and drafted the manuscript. SZ conducted the experiments and supervised the study. YY collected and analyzed the data. QL contributed to the methodology and analyzed the data. CX edited the manuscript. All authors read and approved the final manuscript.

## Conflict of Interest

The authors declare that the research was conducted in the absence of any commercial or financial relationships that could be construed as a potential conflict of interest.

## Supplementary Material

Supplementary Figure 1 *NEAT1* overexpression had no effect on cell viability and apoptosis in ARPE-19 cells. **(A)** The influence of enforced *NEAT1* expression on the viability of ARPE-19 cells was illustrated by CCK-8 assay. **(B)** The impact of *NEAT1* overexpression on the apoptosis of ARPE-19 cells was demonstrated by flow cytometry assay. ns: not significantly.

Supplementary Figure 2 NEAT1 silencing increased the enrichment of LASP1 by miR-3619-5p in Y-79 and SO-RB50 cells. **(A)** RNA pull-down assay was carried out to detect the amount of LASP1 enriched by miR-3619-5p in Y-79 and SO-RB50 cells.

Supplementary Figure 3 The diagram of mechanism of *NEAT1* in regulating RB process.

Supplementary Table 1 Correlation between *NEAT1* expression and clinical clinicopathological parameters of RB.
