# Supplementary figures and images for: LncRNA NEAT1 Knockdown Inhibits Retinoblastoma Progression by miR-3619-5p/LASP1 Axis
Source: Front Genet. 2020 Nov 17;11:574145. doi: 10.3389/fgene.2020.574145 (PMC7705249; doi:10.3389/fgene.2020.574145)

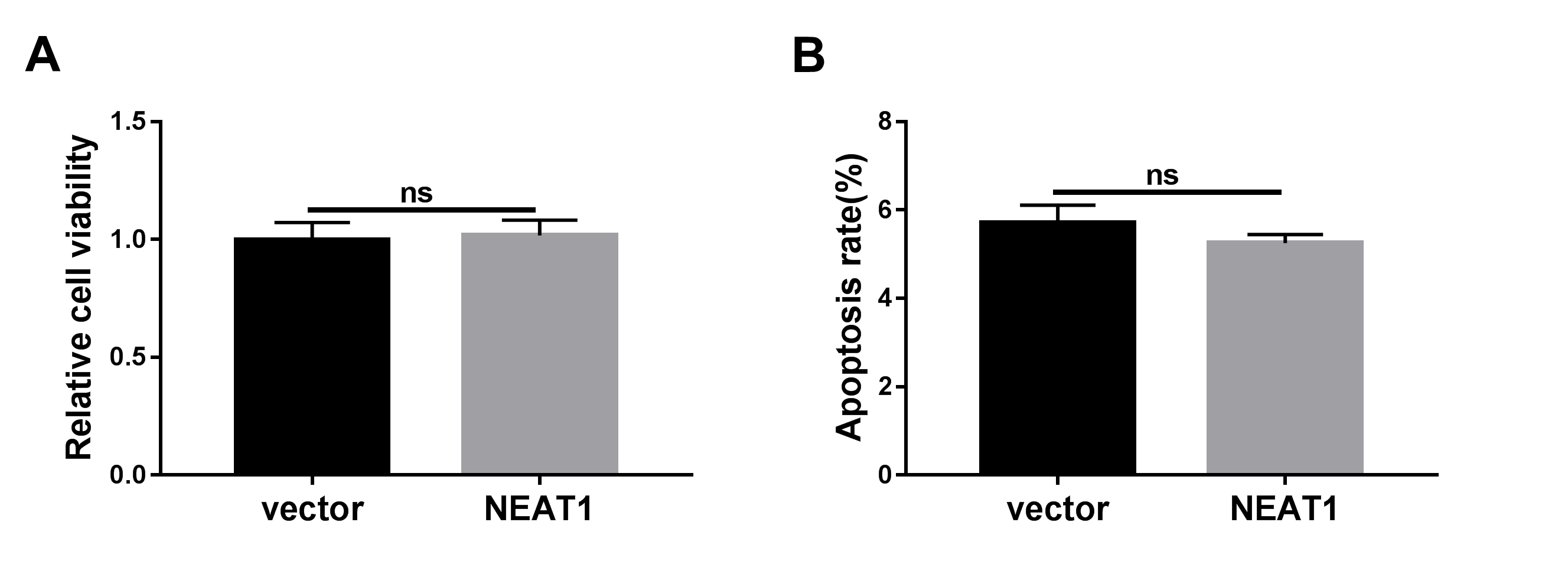

Supplement: Supplementary Figure 1 — NEAT1 overexpression had no effect on cell viability and apoptosis in ARPE-19 cells. (A) The influence of enforced NEAT1 expression on the viability of ARPE-19 cells was illustrated by CCK-8 assay. (B) The impact of NEAT1 overexpression on the apoptosis of ARPE-19 cells was demonstrated by flow cytometry assay. ns: not significantly. [file Image_1.TIF]

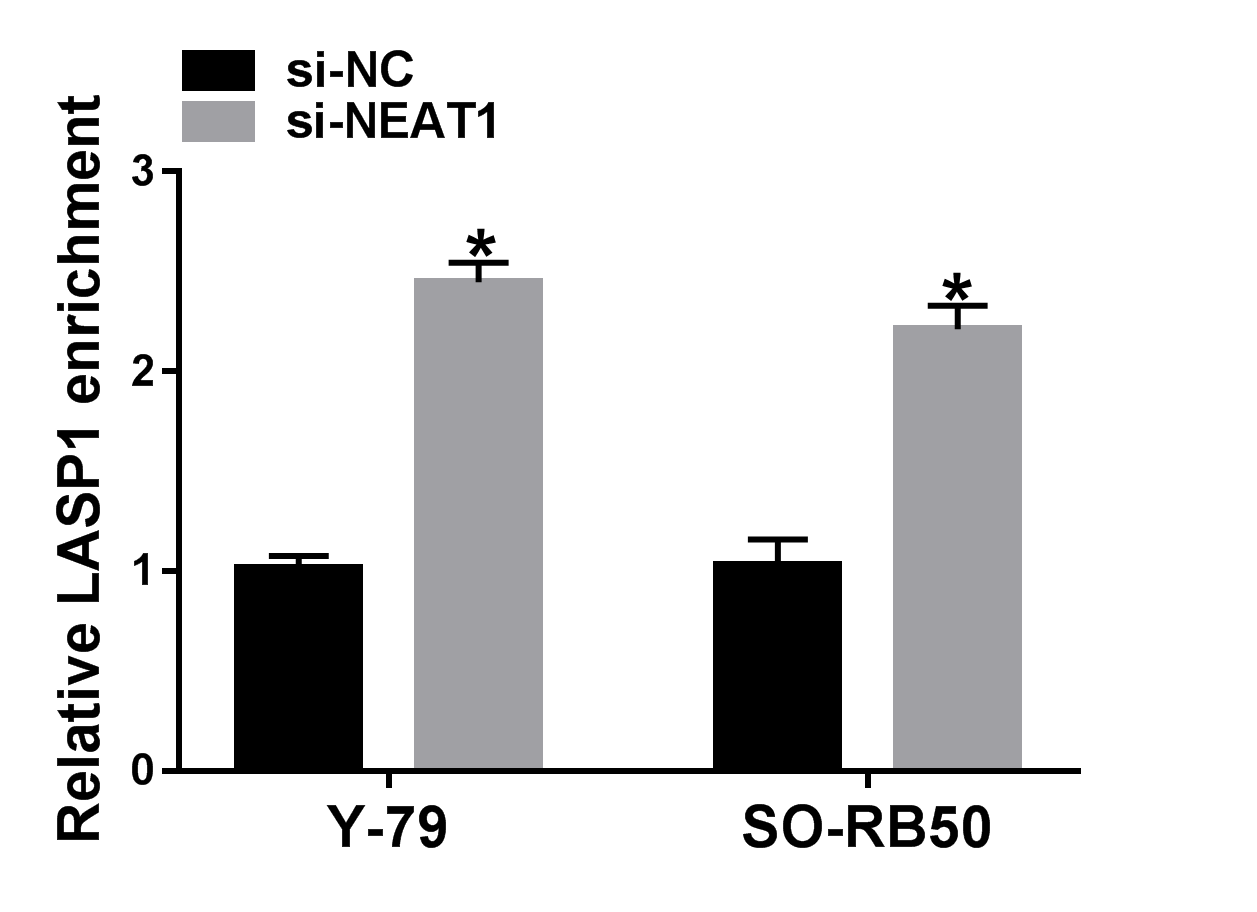

Supplement: Supplementary Figure 2 — NEAT1 silencing increased the enrichment of LASP1 by miR-3619-5p in Y-79 and SO-RB50 cells. (A) RNA pull-down assay was carried out to detect the amount of LASP1 enriched by miR-3619-5p in Y-79 and SO-RB50 cells. [file Image_2.TIF]

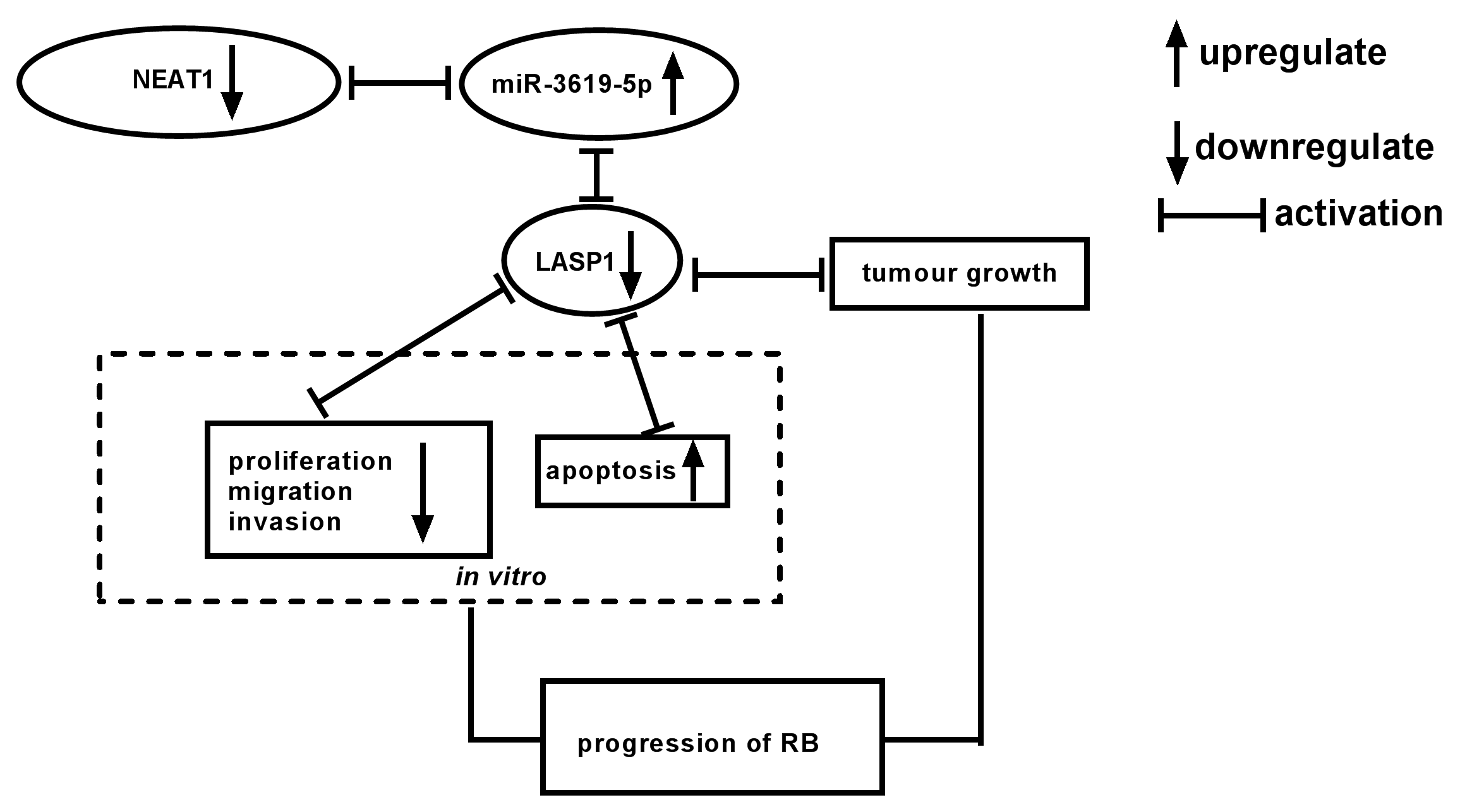

Supplement: Supplementary Figure 3 — The diagram of mechanism of NEAT1 in regulating RB process. [file Image_3.TIF]
