# Supplementary material for: LncRNA NEAT1 Knockdown Inhibits Retinoblastoma Progression by miR-3619-5p/LASP1 Axis
Source: Front Genet. 2020 Nov 17;11:574145. doi: 10.3389/fgene.2020.574145 (PMC7705249; doi:10.3389/fgene.2020.574145)
Supplement: Supplementary Table 1 — Correlation between NEAT1 expression and clinical clinicopathological parameters of RB. [file Table_1.DOCX]

Table S1. Correlation between NEAT1 expression and clinical clinicopathological parameters of RB

| Parameter | Case | NEAT1 expression | | *P* value |
| --- | --- | --- | --- | --- |
|  |  | Low(n=20) | High(n=20) |  |
| Age (years) |  |  |  | 0.527 |
| ≤ 2 | 20 | 11 | 9 |  |
| > 2 | 20 | 9 | 11 |  |
| Gender |  |  |  | 0.744 |
| Female | 15 | 8 | 7 |  |
| Male | 25 | 12 | 13 |  |
| Tumor size |  |  |  | 0.025* |
| ≤15 cm | 17 | 12 | 5 |  |
| >15 cm | 23 | 8 | 15 |  |
| Degree of differentiation |  |  |  | 0.525 |
| Well and moderately | 18 | 8 | 10 |  |
| Poorly | 22 | 12 | 10 |  |
| Choroidal invasion |  |  |  | 0.001** |
| NO | 21 | 16 | 5 |  |
| YES | 19 | 4 | 15 |  |
| Optic nerve invasion |  |  |  | <0.001*** |
| NO | 23 | 18 | 5 |  |
| YES | 17 | 2 | 15 |  |

RB: Retinoblastoma; **P*<0.05, ***P*<0.01 and ****P*<0.001. Significant differences were compared with Chi-square test
